# Impact of SARS-CoV-2 Pandemic on Neuroinfectious Etiology in the Lazio Region: Evidence from Laboratory-Based Surveillance Analysis

**DOI:** 10.3390/v18080883

**Published:** 2026-08-12

**Authors:** Martina Rueca, Sara Leone, Maria Beatrice Valli, Gabriella De Carli, Gaetano Maffongelli, Eleonora Lalle, Federica Forbici, Maria Concetta Fusco, Alessandra Amendola, Lavinia Fabeni, Maria Letizia Giancola, Alessandro Agresta, Tommaso Ascoli Bartoli, Emanuele Nicastri, Fabrizio Maggi, Francesco Vairo

**Affiliations:** 1 Laboratory of Virology and Laboratories of Biosecurity, National Institute for Infectious Diseases ‘Lazzaro Spallanzani’—IRCCS, 00149 Rome, Italy; martina.rueca@inmi.it (M.R.); federica.forbici@inmi.it (F.F.); alessandra.amendola@inmi.it (A.A.);; 2 Clinical Epidemiology and Biostatistics Unit, Department of Epidemiology, Preclinical Research and Advanced Diagnostic, National Institute for Infectious Diseases ‘Lazzaro Spallanzani’—IRCCS, 00149 Rome, Italy; 3 Regional Service for Surveillance and Control of Infectious Diseases (SERESMI), National Institute for Infectious Diseases ‘Lazzaro Spallanzani’—IRCCS, 00149 Rome, Italy; 4 Clinical Department, National Institute for Infectious Diseases ‘Lazzaro Spallanzani’—IRCCS, 00149 Rome, Italy

**Keywords:** meningo-encephalitis, molecular diagnostics, SARS-CoV-2, enterovirus

## Abstract

The mitigation measures adopted to reduce the spread of COVID-19 have not only impacted the transmission of respiratory infections but also other infectious diseases. In this study, we explored the effect of the pandemic on the epidemiology of Central Nervous System (CNS) infections by conducting a retrospective analysis of diagnostic data obtained from cerebrospinal fluids (CSFs) collected before, during, and after the pandemic from patients with acute-suspected neuro-infectious syndrome. The samples were analyzed using a meningitis/encephalitis molecular syndromic panel. A total of 4203 CSFs were split into three groups: pre-pandemic (August 2018–February 2020), pandemic (March 2020–March 2022) and post-pandemic (April 2022–March 2024). Each pathogen was statistically assessed individually, comparing one group vs. another. Results showed that viral pathogens were the most frequently detected across all groups; however, *Streptococcus pneumoniae* was the single most identified pathogen. Comparison of the pre-pandemic and pandemic groups by statistical analysis showed a significant reduction in neuro-infections caused by enterovirus, S. *pneumoniae* and N. *meningitidis*, while the other pathogens were not affected. In conclusion, a reduction in CNS infections caused by respiratory and close-contact-transmitted pathogens, including both viral and bacterial agents, was observed during the period when COVID-19 containment measures were in effect.

## 1. Introduction

Severe acute respiratory syndrome coronavirus 2 (SARS-CoV-2), the causative agent of coronavirus disease (COVID-19), was first identified in Wuhan, Hubei, China, in late December 2019. Only one month later, the World Health Organization (WHO) declared a Public Health Emergency of International Concern (PHEIC) [1]. COVID-19 spread rapidly, causing a global pandemic that lasted for more than two years; as of August 2025, there have been over 778 million cases and 7 million deaths [2].

To cope with the spread of the infection and due to the lack of a specific vaccine and therapy, governments implemented large-scale non-pharmaceutical interventions (NPIs), including lockdowns, mandatory mask-wearing, and social distancing. These measures led to unprecedented human behavioral changes that have had a profound impact not only on the SARS-CoV-2 spreading but also on the etiology and transmission dynamics of numerous other pathogens in many regions worldwide [3,4,5].

Several studies have shown that the widespread adoption of NPIs led to a significant decline in the prevalence of endemic gastrointestinal and respiratory infections, including norovirus, rhinovirus, respiratory syncytial virus (RSV) and influenza viruses [6,7,8]. Surveillance data from multiple countries reported an unprecedented decrease in laboratory-confirmed cases of influenza and RSV, with some areas observing near-complete absence of the typical seasonal epidemics. In particular, influenza faced a huge decline in hospitalization rates and mortality during the pandemic [9].

Similarly, a significant decline was observed in the incidence of certain gastrointestinal infections, particularly those of viral etiology that are transmitted through the fecal–oral route. This is likely attributable to improved hygiene practices and reduced person-to-person contact [8]. Furthermore, the decrease in routine health visits and the focus on SARS-CoV-2 testing and treatment have affected the reporting data of other infectious diseases.

This shift in health priorities has significantly slowed surveillance efforts to control the transmission of infections such as HIV, sexually transmitted infections, and tuberculosis. Regarding the latter, the trend of declining incidence and mortality observed in many parts of the world has been drastically reversed by the pandemic, taking global tuberculosis control back by roughly 10 years [10,11].

Italy was the first European country to be hit by COVID-19: the first autochthonous case was detected on 21 February 2020 in Lombardy, a region in northern Italy, where the infection had been circulating undetected for weeks [12,13]. Soon after, a major outbreak occurred in the region, finding it completely unprepared despite having a strong health care system. The infection spread rapidly with a sharp increase in severe pneumonia cases, quickly overwhelming hospitals, especially intensive care units. Due to concerns that the speed of COVID-19 transmission might exceed the national healthcare system’s capacity, the Government imposed a very strict lockdown and movement restrictions on 9 March, two days before the WHO declared COVID-19 a global pandemic. The national decree banned public gatherings, closed schools, universities, and non-essential businesses, and suspended events; citizens were only allowed to leave their homes for work, health reasons, or essential needs. Furthermore, measures have been introduced such as the requirement to wear a mask even outdoors at various stages of the pandemic, as well as restrictions on travel and movement between different regions of the country. Suddenly, major social activities and interpersonal contacts across the country were markedly reduced. The Italian government adopted a centralized pandemic response, implementing some of the most stringent containment measures in Europe, particularly during the first wave. As a consequence of this stringent national pandemic response, surveillance data from Italian regions such as Lazio revealed an immediate and dramatic decline not only in the transmission of SARS-CoV-2, but also in the incidence of all notifiable infectious diseases (NIDs), with decreases of up to 86%, irrespective of the pathogens and the transmission routes [14].

National countermeasures lasted about two years, with varying levels of stringency depending on the progress of the pandemic, until May 2022.

Other European countries, learning from the Italian experience, were able to implement measures before the case curve. Furthermore, the choices of European governments showed heterogeneous strategies, influenced by different institutional frameworks, healthcare system capacities, and political cultures. These differences produced varied epidemiological and socio-economic outcomes across the continent [15].

In the Lazio region, in 2018, a regional surveillance system of suspected infective meningitis/encephalitis was established, and the National Institute for Infectious Diseases (INMI) Laboratory of Virology was identified as a reference center for the emergency diagnosis of meningitis and encephalitis. Consequently, since then, cerebrospinal fluid (CSF) samples from patients with suspected infectious meningo-encephalitis are sent to our laboratory for diagnosis [16].

In this study, we investigated the impact of the pandemic on the distribution of pathogens causing central nervous system (CNS) infections by retrospectively analyzing CSF data tested with a meningitis/encephalitis molecular syndromic panel from cases presenting an acute neurological syndrome of suspected infectious origin in the Lazio region across pre-pandemic, pandemic, and post-pandemic periods.

## 2. Materials and Methods

### 2.1. Study Design and Sample Collection

This study included CSF specimens collected from patients presenting acute meningo-encephalitis, meningitis and encephalitis of suspected infectious etiology. The patients came from different departments, mainly from the emergency room, intensive care, neonatology, and neurology of hospitals in the Lazio region. Lumbar punctures were performed under sterile conditions, and samples were stored at 2–8 °C prior to analysis. All CSFs were processed within 24 h of collection.

This study was conducted within the framework of the regional surveillance system of acute neurological syndromes of suspected infectious origin, which supports the integration of molecular diagnostics in patients presenting an acute neurological illness, specifically the FilmArray Meningitis/Encephalitis (ME) Panel, into the surveillance and diagnostic pathways for central nervous system infections in the Lazio region [16].

All cases for which CSF samples were analyzed as part of the aforementioned regional surveillance system in the context of urgent clinical evaluation for suspected acute neurological infection were included in the study. Patients were classified as positive cases if at least one of the pathogens in the FilmArray ME Panel was detected.

### 2.2. Molecular Testing with FilmArray ME Panel

Pathogens detection was performed using the FilmArray ME Panel (BioMérieux, Marcy-l’Étoile, France), a fully automated multiplex Polymerase Chain Reaction (PCR) system designed for the simultaneous detection of 14 pathogens (6 bacteria, 7 viruses, and 1 fungus) commonly associated with meningitis and encephalitis: *Escherichia coli K1*, *Haemophilus influenzae*, *Listeria monocytogenes*, *Neisseria meningitidis*, *Streptococcus agalactiae*, *Streptococcus pneumoniae*, cytomegalovirus (CMV), enterovirus (EV), herpes simplex virus 1 (HSV-1), herpes simplex virus 2 (HSV-2), human herpesvirus 6 (HHV-6), human parechovirus (HPeV), varicella zoster virus (VZV) and *Cryptococcus* (*C. neoformans/C. gattii*).

A volume of 200 µL of CSF was loaded into the single-use, self-contained reagent pouch according to the manufacturer’s protocol. The pouch was then inserted into the FilmArray instrument, which automatically performs the following steps: (a) mechanical and chemical cell lysis; (b) nucleic acid extraction and purification; (c) reverse transcription (for RNA viruses); (d) nested multiplex PCR amplification; and (e) detection via melting curve analysis. The total run time for each sample was approximately 90 min.

Each run included internal process controls to monitor sample processing, amplification efficiency, and the presence of PCR inhibitors. Results were considered valid only if all internal controls met predefined acceptance criteria. All outputs were reviewed and validated by trained laboratory personnel.

### 2.3. Data Management and Statistical Analysis

For statistical analyses, the study period (August 2018 to March 2024) was stratified into three distinct phases according to the COVID-19 pandemic, as reported in the Results section.

The results obtained from FilmArray analysis were anonymized and recorded in a secure database. To ensure that statistical analyses were conducted on individual cases, any results of tests performed on the same patient in duplicate or more, even at a later time, within six months from the first diagnosis, were removed from the dataset. In coinfection or multiple-detection cases, each detected pathogen was counted separately and considered as an individual pathogen for positivity rate calculations.

Positivity rate (PR) with 95% confidence intervals (95% CI) of infectious meningo-encephalitis, overall and by pathogen, was estimated for the whole study period and separately for each phase. PR was calculated as the proportion of positive cases among the total number of tested cases.

Comparisons of positivity rates between periods were performed using positivity rate ratios (PRR) with 95% confidence intervals. The following contrasts were evaluated: pandemic vs. pre-pandemic, post-pandemic vs. pandemic, and post-pandemic vs. pre-pandemic.

Descriptive characteristics of infectious meningo-encephalitis-positive cases were summarized overall and across the three study periods. Categorical variables were reported as counts and percentages and compared using Pearson’s chi-squared test or Fisher’s exact test, as appropriate. Continuous variables were summarized as medians and interquartile ranges (IQR) and compared using the Kruskal–Wallis test. When statistically significant differences were identified, pairwise post hoc comparisons between periods were performed. Dunn’s test was used for continuous variables. The Benjamini–Hochberg method was used to correct for the increased risk of type I errors created by multiple tests.

To assess the impact of the COVID-19 pandemic and the subsequent post-pandemic period on monthly infectious meningo-encephalitis cases, an interrupted time series (ITS) analysis was performed using a segmented Poisson regression model [17]. Overdispersion was assessed using the ratio of Pearson’s chi-square statistic to the residual degrees of freedom. As no evidence of overdispersion was detected, Poisson regression models were deemed appropriate. Two interruption time points were set in the analysis: March 2020, when the Italian government implemented the first lockdown, and April 2022, when public health measures to mitigate the spread of COVID-19 were discontinued.

The model included a time variable indicating the number of months since the start of the study period, two dummy variables marking the COVID-19 pandemic and post-pandemic interruptions configured as 0 prior to March 2020 and 1 from March 2020 onward, and as 0 prior to April 2022 and 1 from April 2022 onward, respectively; and the interaction term between each dummy variable and the corresponding time, centered at the respective interruption points.

The model allowed the estimation of the long-term trend in the pre-COVID-19 period and the assessment of both the immediate (level change) and gradual (trend change) effect following the onset of the interruption. The level and trend change following the onset of the COVID-19 pandemic represent the change compared with the expected values if there had been no COVID-19 (counterfactual scenario of no COVID-19 pandemic). Similarly, the level and trend change following the onset of COVID-19 post-pandemic represent the change compared with the expected values if there had been no recovery from COVID-19 (counterfactual scenario of no post-pandemic) [18,19].

Seasonality was controlled by the inclusion of a maximum of two Fourier terms, consisting of pairs of sine and cosine terms. Seasonal components were incorporated if they improved model fit, assessed by the Akaike Information Criterion. To assess autocorrelation, the Ljung–Box test was performed on the model residuals for lags 1 to 3. The third-order lag was considered appropriate according to our outcome, which identified disease transmission dynamics as a primary driver for lag choice [18,20,21].

We estimated the COVID-19 period trend by adding together the coefficients associated with time and the time–COVID-19 interaction. We estimated the post-pandemic period trend by adding together the coefficients associated with time and the time–COVID-19 and time–post-pandemic interactions. A counterfactual scenario in which the COVID-19 pandemic did not occur was estimated by setting the COVID-19 pandemic and post-pandemic dummy to 0. An offset term based on the monthly number of tested cases was included in the model. Subgroup ITS analyses were conducted as secondary analyses to further characterize temporal patterns within selected pathogens with sufficient frequency and temporal distribution of positive cases to support reliable model estimation. Given the exploratory nature of these analyses, no adjustment for multiple comparisons was applied.

The results of the ITS models were reported as PRR, with 95% CI and *p*-values. The trend change was also reported.

A *p*-value less than 0.05 was considered statistically significant. All analyses were conducted using R version 4.2.1.

## 3. Results

From August 2018 to March 2024, 4203 CSF cases with acute neurological syndrome of suspected infectious meningitis/encephalitis received at the Laboratory of Virology of INMI in Rome were analyzed during the study. The study period was divided into three distinct periods according to the COVID-19 pandemic: the PRE period (from August 2018 to February 2020), the DURING period (from March 2020 to March 2022), and the POST period (from April 2022 to March 2024).

**Figure 1:**
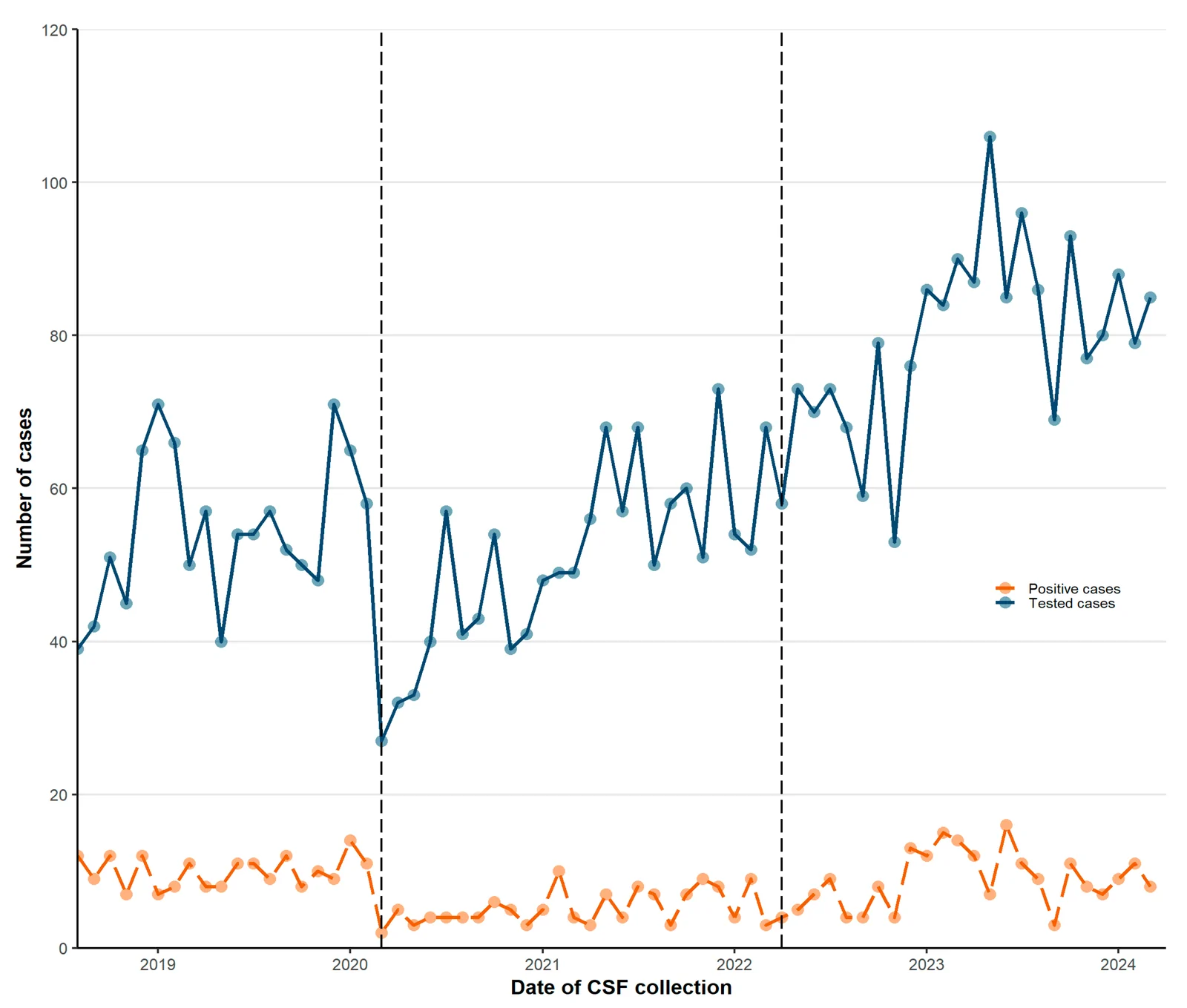
Monthly tested and positive cases across the study populations. August 2018–March 2024.

A total of 1035 CSFs were analyzed in the PRE period, 1268 CSFs in the DURING period, and 1900 in the POST period. Figure 1 shows the number of CSFs from patients with suspected acute neuroinfectious syndrome tested monthly and those that tested positive for one or more pathogens across the study population in the three examined periods.

Overall, the positivity rate of CSF samples decreased during the pandemic compared with the pre-pandemic period. The rate dropped from 18.26% (95% CI 15.95–20.75) pre-pandemic to 10.33% (95% CI 8.71–12.14) during the pandemic, corresponding to a 43% reduction in positivity rate (PRR 0.57, 0.46–0.70). A partial rebound was observed in the post-pandemic period when the rate reached 11.11% (95% CI 9.73–12.60), while it remained lower than the pre-pandemic level (PRR vs. pre-pandemic 0.61, 0.51–0.73). As the pathogens causing infection, viruses were the most frequently found (10.67%, 7.10%, and 6.21% in the PRE, DURING and POST periods, respectively).

Regarding individual pathogens, the positivity rate varies differently in the three periods. In both PRE and POST periods, viruses were the most frequently identified pathogens in CFSs overall, whereas *S. pneumoniae* was the most frequently identified single pathogen when individual microorganisms were analyzed. More specifically, in the PRE period, *S. pneumoniae* infections were the most representative (4.44%), followed by EV infections (3.67%), HSV-1, VZV, HHV-6 and *N. meningitidis* infections (2.03%, 1.93%, 1.93%, and 1.16% respectively).

**Figure 2:**
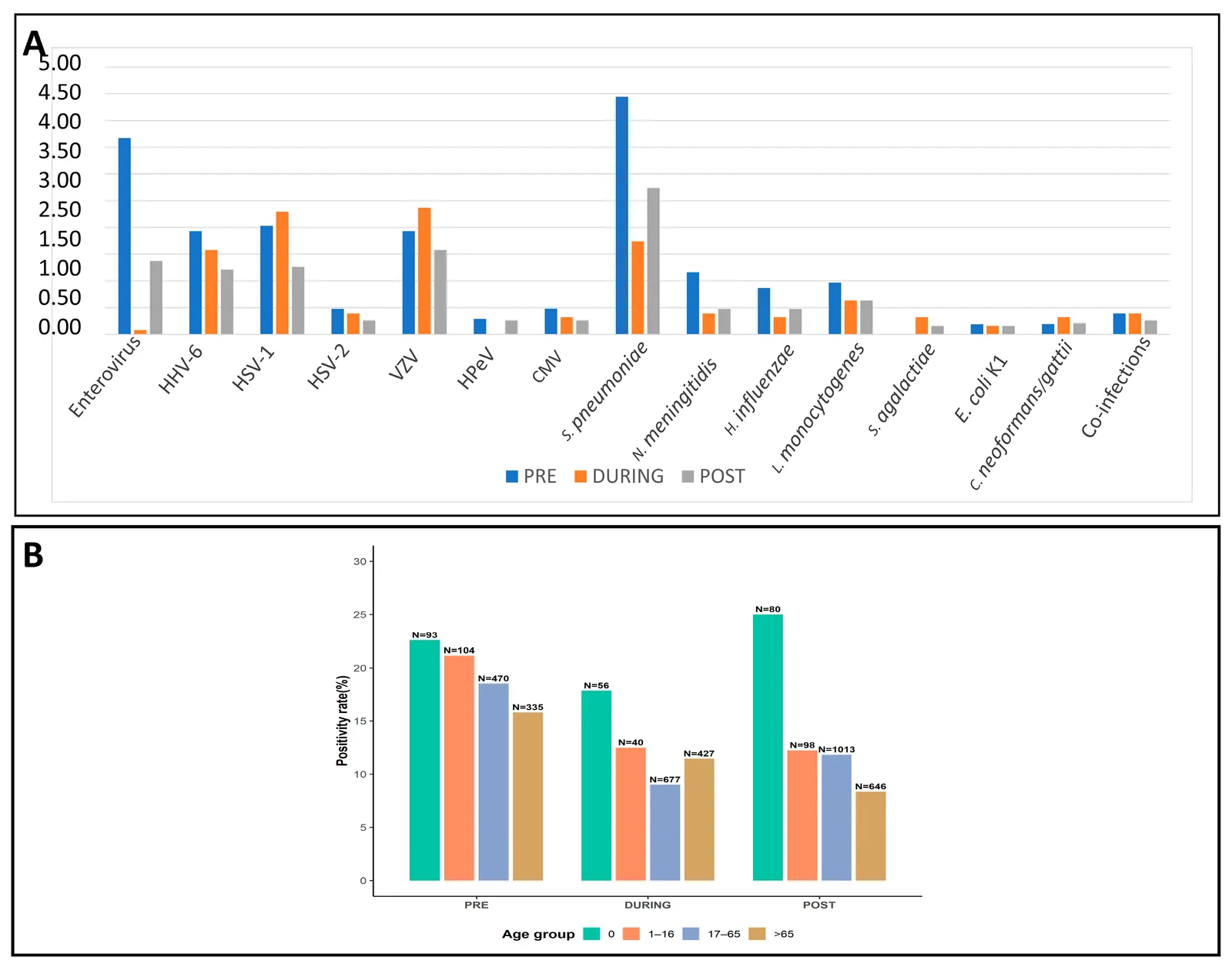
(**A**) Proportion of positive cases by pathogen, defined as positive detections for that specific pathogen over the total number of tested patients. (**B**) Positivity rate by age group, calculated as the number of positive cases divided by the total number of tested cases within each age group for each pandemic period. Numbers above the bars indicate the total number of tested cases.

During the SARS-CoV-2 pandemic, VZV detection increased to 2.37%, followed by HSV-1 with 2.29%. Conversely, the detection rate of *S. pneumoniae* decreased to 1.74%, as did that of EV, which declined to 0.08%. As far as the POST period is concerned, the positive rate for *S. pneumoniae* increased again, reaching 2.74%, whereas the positivity rates of VZV, EV, HSV1 and HHV-6 were 1.68%, 1,37%, 1.37% and 1.37%, respectively, (Figure 2A; Table 1). Of interest, among the most frequently detected pathogens, the positivity rate of HSV-1 did not change significantly during the pandemic compared with the pre-pandemic period (PRR 1.13, 0.65–1.96); however, a significant 42% reduction was observed in the post-pandemic period compared with the pandemic period (PRR 0.58, 0.34–0.98).

The positivity rate of *S. pneumoniae* infection decreased significantly during the pandemic compared with the pre-pandemic period (4.4% vs. 1.7%; PRR 0.39, 0.24–0.64). In the post-pandemic period, the rate was 2.7%, remaining significantly lower than the pre-pandemic period (PRR 0.62, 0.42–0.91).

The positivity rate of EV infection showed a sharp decline during the pandemic, falling from 3.67% in the pre-pandemic period to 0.08% during the pandemic (PRR 0.02, 0–0.16). In the post-pandemic period, the rate increased significantly compared with the pandemic period (PRR 17.35, 2.36–127.7); however, it remained significantly lower than pre-pandemic levels (PRR 0.37, 0.23–0.61).

A significant reduction in the positivity rate of *N. meningitidis* infection was observed during the pandemic and in the post-pandemic periods compared with the pre-pandemic period (PRR 0.34, 0.12–0.96; PRR 0.41, 0.17–0.97, respectively).

When considering all the *Herpesviridae* viruses, the positivity rate in the analyzed CSFs remained stable during the pandemic compared with the pre-pandemic period (PRR 0.99, 0.73–1.35), but significantly decreased in the post-pandemic period relative to both the pre-pandemic and during-pandemic periods (PRR 0.70, 0.52–0.95; PRR 0.71, 0.53–0.94, respectively).

For other pathogens, no statistically significant differences were observed across the study periods (Figure 2A; Table 1). Among cases testing positive for infectious meningo-encephalitis, 48% were male, with no statistically significant differences across the study periods (*p* = 0.832). Overall, median age was 53 years (IQR 29–69), with statistically significant differences across the three periods (overall *p* = 0.027). Pairwise comparisons between subgroups showed a significantly lower median age in the pre-pandemic period compared with during the pandemic (49 vs. 58 years, p_BH_ < 0.001) and post-pandemic periods (49 vs. 52.5, p_BH_ < 0.001) (Table 2). Of interest, according to age group, across the three periods examined, most positive cases occurred in the age group of infants (under one year old), followed by the other age groups (Figure 2B).

### 3.1. ITS Analysis

Within the interrupted time series (ITS) analysis, assessment of overdispersion using the ratio of Pearson’s chi-square statistic to the residual degrees of freedom showed no evidence of overdispersion in any of the fitted Poisson models (dispersion ratios: 0.78–1.30; all *p* > 0.05). Overall, ITS analysis showed a stable trend in the positivity rate of infectious meningo-encephalitis in the pre-pandemic period (PRR 1.00, 95% CI 0.97–1.02). At the onset of the pandemic, the rate dropped remarkably, with a 42% statistically significant reduction, compared to the counterfactual (PRR 0.58, 0.36–0.93). No statistically significant trend change was observed following pandemic onset (PRR 1.01, *p* = 0.716), and the trend during the pandemic remained stable (PRR 1.00, 0.98–1.03). In April 2022, with the onset of the post-pandemic period, no significant level change was observed (PRR 0.99, 0.64–1.54), compared to the counterfactual. Similarly, no significant trend change was detected (PRR 1.00, *p* = 0.819). In the post-pandemic period, an upward trend of 1% per month in the positivity rate of infectious meningo-encephalitis was recorded, although it was not statistically significant (PRR 1.01, 0.99–1.03).

**Figure 3:**
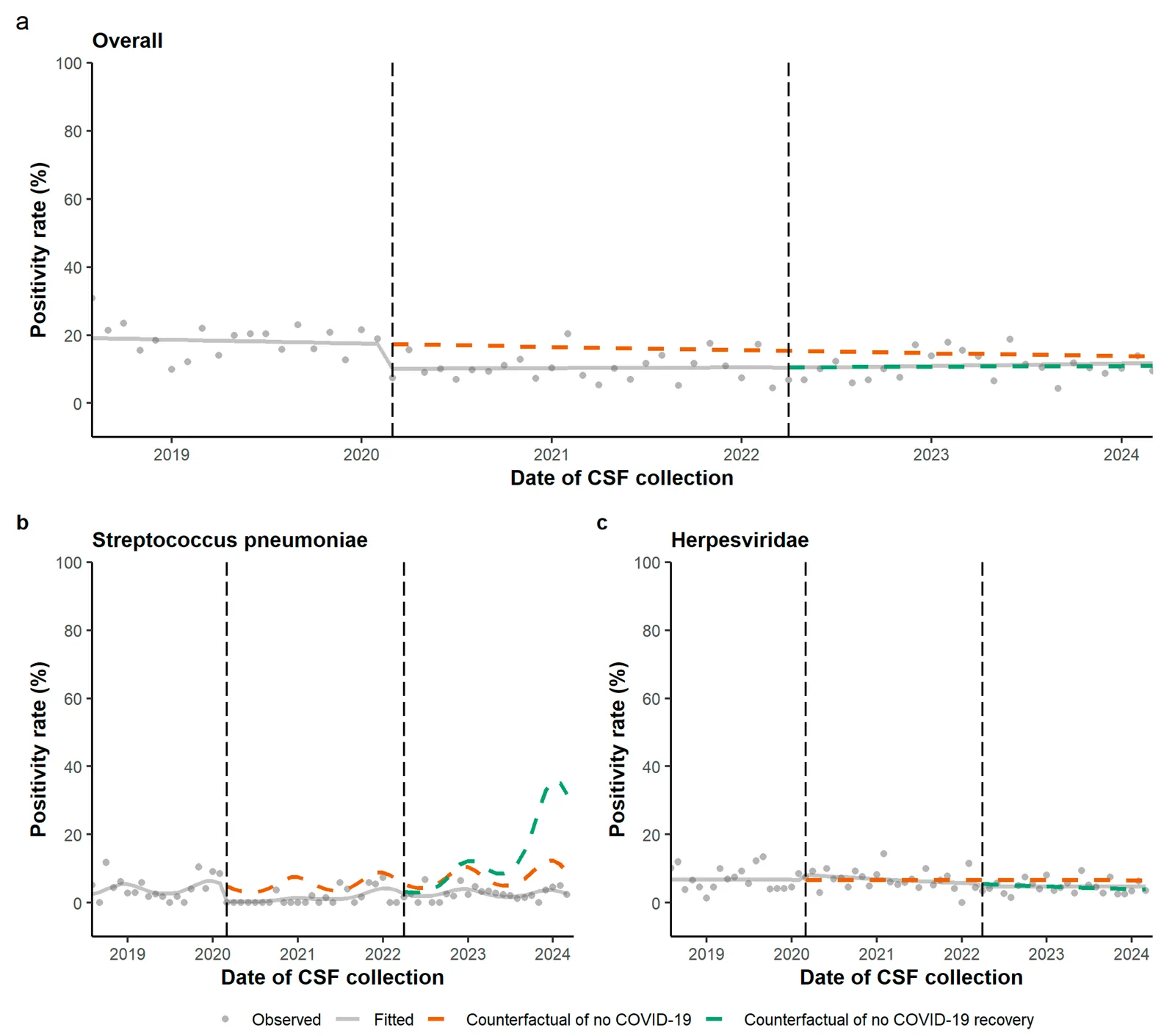
Observed positivity rate and results of ITS analysis overall and by selected microorganisms in the Lazio Region, 1 August 2018–31 March 2024. (**a**) Overall positivity rate. (**b**) Positivity rate for Streptococcus pneumoniae. (**c**) Positivity rate for Herpesviridae. Grey dots represent the observed monthly positivity rates, the grey solid line represents the fitted values from the ITS model, the orange dashed line represents the counterfactual scenario assuming no COVID-19 pandemic, and the green dashed line represents the counterfactual scenario assuming no COVID-19 recovery. Vertical dashed lines indicate the start of the COVID-19 pandemic period and the start of the post-pandemic period.

Pathogen subgroup ITS analyses were conducted for *S. pneumoniae* (120 positive cases), the pathogen with the highest number of detections, and for the *Herpesviridae* group (245 cases), representing the largest biologically coherent viral subgroup. Most of the remaining pathogens were not considered suitable for pathogen-specific ITS analyses because of the low frequency of positive cases, ranging from 7 (*Streptococcus agalactiae* and *Escherichia coli* K1) to 31 (*Listeria monocytogenes*). Furthermore, although enterovirus accounted for 65 positive cases, only one occurred during the pandemic period, resulting in a sparse temporal distribution that precluded reliable model estimation. The exploratory subgroup ITS analysis results broadly mirrored the general pattern, although some differences were observed. With the start of the COVID-19 pandemic, the positivity rate of *S. pneumoniae* fell sharply (PRR 0.09, 0.02–0.35). Although the upward trend change did not reach statistical significance (PRR 1.08, *p* = 0.09), the positivity rate showed a significant increasing trend of 9% per month during the pandemic (PRR 1.09, 1.02–1.18). Following the onset of the post-pandemic period, a significant downward trend change was observed (PRR 0.91, *p* = 0.022). Conversely, the positivity rate of *Herpesviridae* exhibited an increase of 22% at pandemic onset (PRR 1.22, 0.64–2.35), while a downward trend change (PRR 0.98, *p* = 0.569) and a decreasing trend of 2% per month were observed during the whole COVID-19 period (PRR 0.98, 0.95–1.01), although all variations were not statistically significant. Across all fitted models, no autocorrelation was detected. Autocorrelation (ACF) and partial autocorrelation (PACF) plots of the model residuals were provided in the Table S1. Seasonality was accounted for using one Fourier term only in the *S. pneumoniae* model, where its inclusion improved model fit (Table 3 and Figure 3).

### 3.2. Enterovirus CNS Infections: Trend of Circulation

**Figure 4:**
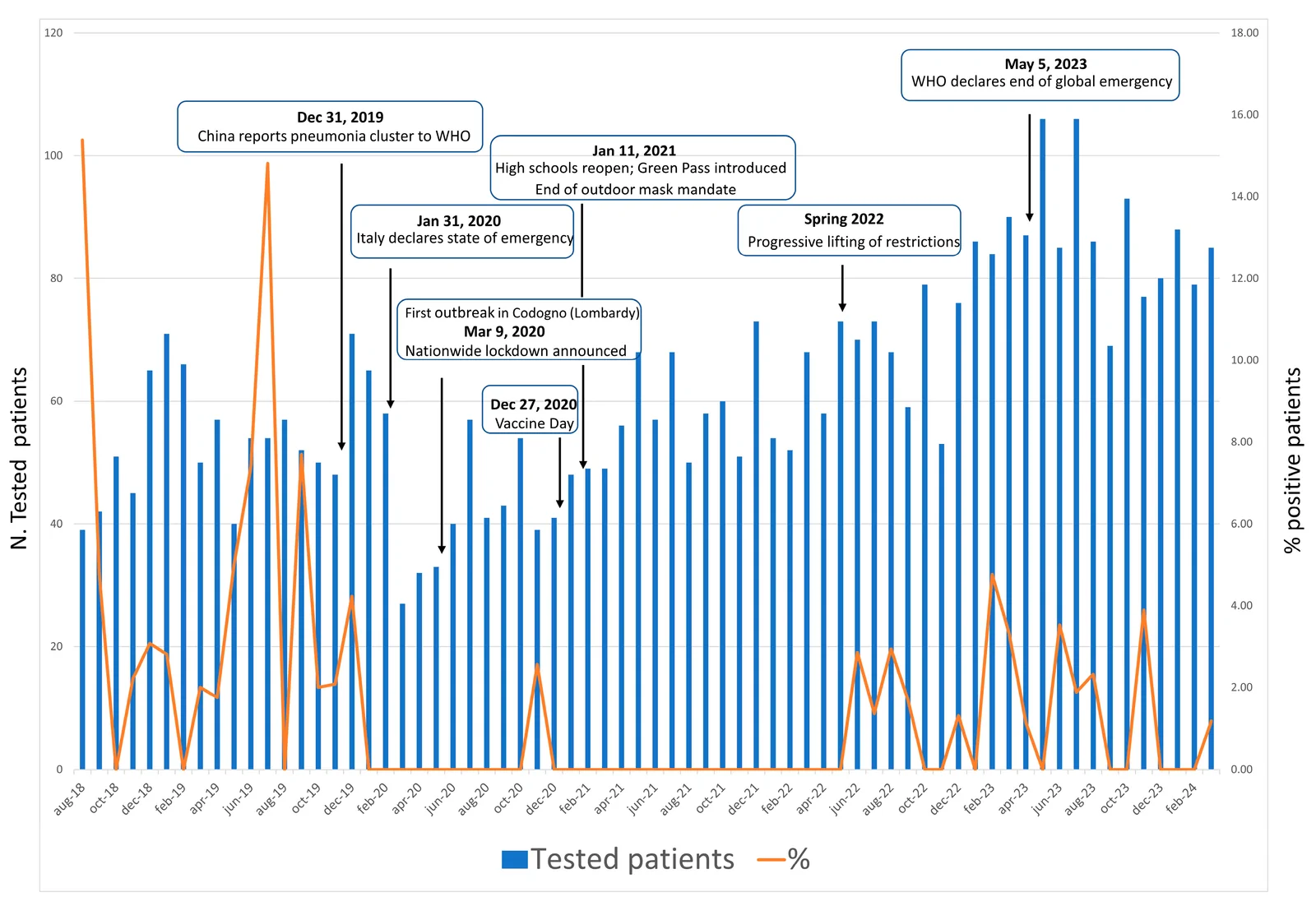
Trend of EV infections. Blue bars indicate tested patients for EV diagnosis at the INMI Laboratory of Virology, while the orange line defines the percentage of positive patients.

We therefore proceeded to analyze the trend of EV circulation during the study period in greater detail. Figure 4 shows the number of EV-positive patients compared with the total number of patients tested at the INMI Laboratory of Virology. As shown in the figure, the number of tested cases progressively rose from August 2018 to June 2024, with a decrease observed during the early phases of the SARS-CoV-2 pandemic (2020–2021), shortly following the implementation of the national lockdown. Regarding positive cases, their number decreased dramatically in the years affected by the pandemic and by the resulting restrictive measures of social distancing (2020–2022), while they increased again since June 2022, when social distancing was progressively lifted, showing a seasonal pattern typical of EV infection, which is more prevalent in the spring and summer seasons.

## 4. Discussion

The COVID-19 pandemic has profoundly reshaped the global landscape of infectious diseases, not only through its direct impact but also by altering the epidemiology and transmission dynamics of numerous other pathogens [3,4,5,6,7,8,9]. Our study, including over a five-year period and encompassing the pre-pandemic (PRE), pandemic (DURING), and post-pandemic (POST) period, highlights substantial changes in the prevalence and distribution of pathogens associated with neurological infections, such as meningitis and encephalitis. These changes may be associated, at least in part, with the implementation and subsequent relaxation of NPIs, such as social distancing, lockdowns, school closures, and enhanced hygiene practices. However, the extent to which these measures contributed to the observed trends remains uncertain.

One of the most impactful observations in our dataset is the significant reduction in the overall positivity rate for CSFs of suspected cases during the pandemic phase (10.33%), compared to the pre-pandemic period (18.26%). This decrease may be linked to multiple SARS-CoV-2-related factors, including social distancing, mask-wearing, enhanced hygiene practices, and changes in routine diagnostics and surveillance activities. Potential sources of bias should be acknowledged, including changes in clinical priorities, limited access to hospital care, and modifications in diagnostic workflows during the pandemic. Nevertheless, the relatively comparable number of samples analyzed across the PRE, DURING and POST periods may partially mitigate concerns regarding the robustness of the observed trends.

Similar patterns have been reported worldwide for other infectious diseases, particularly respiratory and gastroenteric infections caused by pathogens such as influenza, RSV, and norovirus, all of which exhibited marked declines during periods of stringent NPIs [5,9,22].

The reduction in endemic infections observed during the COVID-19 pandemic was followed by a resurgence of common pathogens in the post-pandemic period (e.g., *S. pneumoniae* and EV), as previously described by other authors both in national and international settings [23,24]. One hypothesis is that this phenomenon may be related to the concept of “immunity debt”, whereby reduced exposure to common respiratory and community-acquired pathogens during COVID-19 mitigation measures may have increased the proportion of susceptible individuals and temporally reduced the population-level immunity, particularly among children and infants, thereby contributing to the subsequent rebound of infections [25]. This pattern is evident in the data presented here, where the majority of positive cases across all periods were in patients under one year of age, underscoring the vulnerability of this demographic group to CNS infections [15]. 

From a pathogen-specific perspective, the predominance of viral etiologies during all phases confirms previous literature emphasizing the high prevalence of viral agents in neuroinfectious diseases [26]. Notably, the positivity rate of EV infection substantially dropped during the pandemic (from 3.7% of PRE to 0.1% of DURING with PRR 0.02, 0–0.16); moreover, in the POST period, the rate increased significantly compared with the during-pandemic period (PRR 17.35, 2.36–127.7). This pattern aligns with surveillance reports from other countries and confirms the sensitivity of EV transmission to behavioral and environmental changes [27]. However, even though EV cases increased to 1.3% in the post-pandemic period, they remained significantly lower than in the pre-pandemic period.

Interestingly, while bacterial pathogens such as *S. pneumoniae* showed a marked drop at the onset of the pandemic (PRR 0.09, 0.02–0.35), followed by an increasing trend of 9.0% per month during the pandemic (PRR 1.09, 1.02–1.18). This trend likely reflects the combined influence of reduced person-to-person transmission during NPIs and subsequent increased exposure as restrictions were lifted. Additionally, the non-statistically significant increase in *Herpesviridae* positivity observed at the onset of the pandemic raises questions about a possible role of stress-related factors; further investigations are therefore warranted to explore this hypothesis.

While the use of the FilmArray ME Panel allowed for rapid, standardized, and multiplexed detection of a wide range of pathogens, a limitation of our study lies in the diagnostic appropriateness. Several factors, including changes in diagnostic demands, reduced access to healthcare during the pandemic, and the high frequency of neurological manifestations (reported in more than 30% of COVID-19 cases) [28], may have introduced a bias in the clinical threshold for performing lumbar punctures.

In addition, the molecular detection of Betaherpesvirinae, such as HHV-6 and CMV, in cerebrospinal fluids by the FilmArray ME Panel does not necessarily indicate a causative neuroinfectious agent, as positive results may reflect latent infection, viral reactivation, or incidental detection. This is particularly relevant for HHV-6, as individuals with chromosomally integrated HHV-6 (ciHHV-6) may yield positive PCR results in the absence of active viral replication or disease [29,30]. Therefore, these findings should be interpreted in the context of the patient’s overall clinical presentation and supporting laboratory and radiological evidence.

Nonetheless, our findings highlight the importance of maintaining robust and dynamic infectious disease surveillance systems that can adapt to sudden epidemiological shifts. The COVID-19 pandemic has demonstrated how global health concerns can influence pathogen circulation, necessitating coordinated surveillance and preparedness strategies that extend beyond a single disease focus.

## 5. Conclusions

In conclusion, this study provides clear evidence that the COVID-19 pandemic altered the epidemiology of CNS infections in the Lazio region. The observed changes in pathogen prevalence and seasonal dynamics underscore the need for sustained vigilance in post-pandemic infectious disease monitoring. Future research should explore the long-term implications of these shifts on population immunity, vaccine strategies, and clinical management of neuroinfectious diseases.

## Figures and Tables

**Table 1:** Positivity rates overall and positivity rate ratios of neuroinfectious diagnostic data over the study period, by period and by microorganism, 1 August 2018–31 March 2024.

	Over the Study Period	Pandemic vs. Pre-Pandemic	Post-Pandemic vs. Pandemic	Post-Pandemic vs. Pre-Pandemic
	PR (95% CI) ^1^	PRR (95% CI) ^2^	PRR (95% CI) ^2^	PRR (95% CI) ^2^
Overall	12.63	0.57	1.07	0.61
	(11.64–13.68)	(0.46–0.7)	(0.87–1.32)	(0.51–0.73)
CMV	0.33	0.65	0.83	0.54
	(0.18–0.56)	(0.18–2.43)	(0.22–3.1)	(0.16–1.88)
*L. monocytogenes*	0.74	0.65	1.08	0.71
	(0.5–1.05)	(0.26–1.65)	(0.45–2.61)	(0.31–1.61)
HSV1	1.78	1.13	0.58	0.65
	(1.41–2.23)	(0.65–1.96)	(0.34–0.98)	(0.36–1.15)
*S. pneumoniae*	2.86	0.39	1.58	0.62
	(2.37–3.4)	(0.24–0.64)	(0.96–2.58)	(0.42–0.91)
*S. agalactiae*	0.17	--	0.5	--
	(0.07–0.34)		(0.11–2.23)	
*E coli* K1	0.17	0.82	1	0.82
	(0.07–0.34)	(0.12–5.78)	(0.17–5.98)	(0.14–4.88)
*C. neoformans gattii*	0.24	1.63	0.67	1.09
	(0.11–0.44)	(0.3–8.9)	(0.17–2.66)	(0.2–5.94)
HHV6	1.57	0.82	0.87	0.71
	(1.22–1.99)	(0.44–1.51)	(0.49–1.55)	(0.4–1.26)
Enterovirus	1.55	0.02	17.35	0.37
	(1.2–1.97)	(0–0.16)	(2.36–127.7)	(0.23–0.61)
*H. influenzae*	0.52	0.36	1.5	0.54
	(0.33–0.79)	(0.11–1.17)	(0.46–4.87)	(0.22–1.37)
HSV2	0.36	0.82	0.67	0.54
	(0.2–0.59)	(0.24–2.81)	(0.19–2.3)	(0.16–1.88)
*N. meningitidis*	0.62 (0.4–0.91)	0.34	1.2	0.41
		(0.12–0.96)	(0.4–3.58)	(0.17–0.97)
HPeV	0.19	--	--	0.91
	(0.08–0.37)			(0.22–3.79)
VZV	1.95	1.22	0.71	0.87
	(1.55–2.42)	(0.70–2.14)	(0.43–1.17)	(0.5–1.52)
*Herpesviridae* (CMV + HSV1 + HHV6 + HSV2 + VZV)	5.83	0.99	0.71	0.7
	(5.14–6.58)	(0.73–1.35)	(0.53–0.94)	(0.52–0.95)

^1^ PR: positivity rate, ^2^ PRR: positivity rate ratio.

**Table 2:** Distribution of infectious meningo-encephalitis cases across study periods.

		Period			
Variable	Overall	Pre-Pandemic	Pandemic	Post-Pandemic	*p*-Value ^2^
	N = 531 (100%) ^1^	N = 189 (36%) ^1^	N = 131 (25%) ^1^	N = 211 (40%) ^1^	
Sex					0.832
Male	255 (48.0%)	90 (47.6%)	58 (44.3%)	107 (50.7%)	
Female	235 (44.3%)	84 (44.4%)	63 (48.1%)	88 (41.7%)	
Unknown	41 (7.7%)	15 (7.9%)	10 (7.6%)	16 (7.6%)	
Age	53.0 (29.0, 69.0)	49.0 (20.0, 68.0) *	58.0 (37.0, 73.0) ^†^	52.5 (31.0, 67.0)	0.027
Missing	17 (3.2)	6 (3.2)	6 (4.6)	5 (2.4)	
Age-group					0.033
<1 year	51 (9.6%)	21 (11.1%)	10 (7.6%)	20 (9.5%)	
1–16	39 (7.3%)	22 (11.6%)	5 (3.8%)	12 (5.7%)	
17–65	268 (50.5%)	87 (46.0%)	61 (46.6%)	120 (56.9%)	
>65	156 (29.4%)	53 (28.0%)	49 (37.4%)	54 (25.6%)	
Unknown	17 (3.2%)	6 (3.2%)	6 (4.6%)	5 (2.4%)	

^1^ n (%); Median (Q1, Q3). ^2^ Pearson’s Chi-squared test; Kruskal–Wallis rank sum test; Fisher’s Exact Test. * *p*-value < 0.05, comparison between the pre-pandemic and the during pandemic periods (adjusted with Benjamini–Hochberg method). ^†^ *p*-value < 0.05, comparison between the pre-pandemic and the post-pandemic periods (adjusted with Benjamini–Hochberg method).

**Table 3:** Interrupted time series analysis estimating the impact of COVID-19 and POST COVID-19 periods on Neuroinfectious diagnostic data overall and by selected microorganisms; 1 August 2018–31 March 2024.

	Pre-Pandemic Period		Pandemic Period						Post-Pandemic Period					
	Trend		Level Change ^2^		Trend Change ^2^		Trend		Level Change ^3^		Trend Change ^3^		Trend	
	PRR	*p*	PRR	*p*	PRR	*p*	PRR	*p*	PRR	*p*	PRR	*p*	PRR	*p*
	(95% CI)		(95% CI)		(95% CI)		(95% CI)		(95% CI)		(95% CI)		(95% CI)	
Overall	1.00	0.706	0.58	0.025	1.01	0.716	1.00	0.897	0.99	0.957	1.00	0.819	1.01	0.602
	(0.97, 1.02)		(0.36, 0.93)		(0.97,1.04)		(0.98, 1.03)		(0.64, 1.54)		(0.97, 1.04)		(0.99, 1.03)	
*S. pneumoniae* ^1^	1.01	0.605	0.09	0.001	1.08	0.095	1.09	0.017	0.78	0.597	0.91	0.022	0.99	0.714
	(0.96, 1.07)		(0.02, 0.35)		(0.99, 1.18)		(1.02, 1.18)		(0.31, 1.99)		(0.83, 0.98)		(0.95, 1.03)	
*Herpesviridae* ^4^	1.00	0.982	1.22	0.546	0.98	0.569	0.98	0.308	0.86	0.633	1.02	0.490	1.00	0.970
	(0.96, 1.04)		(0.64, 2.35)		(0.93, 1.04)		(0.95, 1.01)		(0.47, 1.59)		(0.95, 1.03)		(0.97, 1.03)	

^1^ Model adjusted for seasonality. ^2^ Compared to counterfactual scenario had there been no COVID-19. ^3^ Compared to counterfactual scenario had there been no COVID-19 recovery. ^4^ CMV + HSV1 + HSV2 + HHV6 + VZV. PRR: Positivity Rate Ratio; CI: Confidence Interval.

## Data Availability

The original contributions presented in this study are included in the article/supplementary material. Further inquiries can be directed to the corresponding author.

## Supplementary Materials

The following supporting information can be downloaded at: https://www.mdpi.com/article/10.3390/v18080883/s1, Table S1: Positivity rate ratios of Neuroinfectious diagnostic data over the study period, by period and by microorganism, 1 August 2018–31 March 2024.
