# Supplementary material for: Impact of SARS-CoV-2 Pandemic on Neuroinfectious Etiology in the Lazio Region: Evidence from Laboratory-Based Surveillance Analysis
Source: Viruses. 2026 Aug 12;18(8):883. doi: 10.3390/v18080883 (PMC13517623; doi:10.3390/v18080883)
Supplement: Supplementary file 1 [file viruses-18-00883-s001.zip › viruses-4400648-supplementary.pdf]

Table S1: Positivity rate ratios of Neuroinfectious diagnostic data over the study period, by period and by microorganism ,1 August 2018 – 31 March 2024

	Period		
	**Pre-pademic**	**During-pandemic**	**Post-pandemic**
	**PR (95% CI)[1]**	**PR (95% CI)[1]**	**PR (95% CI)[1]**
**Overall**	18.26 (15.95 - 20.75)	10.33 (8.71 - 12.14)	11.11 (9.73 - 12.60)
**CMV**	0.48 (0.16 - 1.12)	0.32 (0.09 - 0.81)	0.26 (0.09 - 0.61)
L. monocytogenes	0.97 (0.46 - 1.77)	0.63 (0.27 - 1.24)	0.68 (0.36 - 1.17)
**HSV1**	2.03 (1.26 - 3.08)	2.29 (1.54 - 3.27)	1.32 (0.85 - 1.94)
S. pneumoniae	4.44 (3.27 - 5.88)	1.74 (1.09 - 2.62)	2.74 (2.05 - 3.57)
S. agalactiae	0.00 (0.00 - 0.36)	0.32 (0.09 - 0.81)	0.16 (0.03 - 0.46)
***E coli* K1**	0.19 (0.02 - 0.70)	0.16 (0.02 - 0.57)	0.16 (0.03 - 0.46)
C. neoformans gattii	0.19 (0.02 - 0.70)	0.32 (0.09 - 0.81)	0.21 (0.06 - 0.54)
**HHV6**	1.93 (1.18 - 2.97)	1.58 (0.97 - 2.43)	1.37 (0.90 - 2.00)
**Enterovirus**	3.67 (2.61 - 5.00)	0.08 (0.00 - 0.44)	1.37 (0.90 - 2.00)
H. influenzae	0.87 (0.40 - 1.64)	0.32 (0.09 - 0.81)	0.47 (0.22 - 0.90)
**HSV2**	0.48 (0.16 - 1.12)	0.39 (0.13 - 0.92)	0.26 (0.09 - 0.61)
N. meningitidis	1.16 (0.60 - 2.02)	0.39 (0.13 - 0.92)	0.47 (0.22 - 0.90)
**HPeV**	0.29 (0.06 - 0.84)	0.00 (0.00 - 0.29)	0.26 (0.09 - 0.61)
**VZV**	1.93 (1.18 - 2.97)	2.37 (1.60 - 3.36)	1.68 (1.15 - 2.37)
**Herpesviruses (CMV+HSV1+HHV6+HSV2+VZV)**	6.76 (5.31- 8.47)	6.7 (5.39 - 8.22)	4.74 (3.83 - 5.79)
